# Association of Demographic, Clinical, and Social Determinants of Health With COVID-19 Vaccination Booster Dose Completion Among US Veterans

**DOI:** 10.1001/jamanetworkopen.2022.22635

**Published:** 2022-07-19

**Authors:** Karen H. Seal, Daniel Bertenthal, Jennifer K. Manuel, Jeffrey M. Pyne

**Affiliations:** 1 San Francisco Veterans Affairs (VA) Healthcare System, San Francisco, California; 2 Departments of Medicine and Psychiatry, University of California, San Francisco; 3 Department of Psychiatry, University of California, San Francisco; 4 Mental Health Service, Central Arkansas VA Healthcare System, Little Rock; 5 Department of Psychiatry, University of Arkansas School of Medicine, Little Rock

## Abstract

This cohort study investigates the association of demographic, clinical, and social determinants of health with COVID-19 booster completion among enrollees in the US Veterans Health Administration to identify vulnerable subpopulations.

## Introduction

COVID-19 vaccination markedly decreases serious illness, hospitalization, and mortality due to SARS-CoV-2 infection, but immunity wanes, leaving individuals susceptible to COVID-19.^1^ Thus, the Centers for Disease Control and Prevention recommend vaccine boosters. Some subpopulations have lower rates of primary COVID-19 vaccination than others,^2^ suggesting that increasing numbers of individuals will lack protection against COVID-19 should booster-eligible individuals fail to receive boosters.

Of 6 173 062 US veterans enrolled in the Veterans Health Administration (VHA), 3 949 343 (64.0%) completed primary COVID-19 vaccination and were eligible to receive a booster. We investigated the association of demographic, clinical, and social determinants of health with COVID-19 booster completion to identify vulnerable subpopulations.

## Methods

The VHA electronic health record (EHR) was used to construct a retrospective cohort of 3 578 627 veterans from December 11, 2020 (first Emergency Use Authorization approval and first COVID-19 vaccinations in the VHA), through February 8, 2022 (eFigure in the Supplement). Inclusion criteria were at least 1 VHA outpatient visit and eligibility for the first COVID-19 vaccine booster based on Centers for Disease Control and Prevention–specified intervals from primary series completion. Veterans who received a third dose within 6 weeks of primary vaccination for immunocompromise (eg, receiving chemotherapy) or lacking complete data were excluded. The VHA Central Institutional Review Board approved this study and waived informed consent for EHR review. The study followed the STROBE reporting guideline. The main outcome was receipt of the first COVID-19 booster after primary vaccination within or outside the VHA, as captured in the EHR. Statistical analyses are described in the eMethods in the Supplement. Univariate descriptive statistics determined unadjusted proportions of veterans completing boosters by subgroup; generalized linear models with predictive margins estimated adjusted rates and differences considering potential confounding. Two-sided *P* < .05 indicated statistical significance.

## Results

 Of 3 578 627 eligible VHA enrollees, the mean (SD) age was 65.9 (15.1) years; 8.8% were female and 91.2% were male. In terms of self-reported race and ethnicity (obtained from the EHR as key social determinants of vaccination^3^), 0.6% were American Indian or Alaska Native, 1.4% were Asian, 7.1% were Hispanic, 0.7% were Native Hawaiian or other Pacific Islander, 18.3% were non-Hispanic Black or African American, 0.8% were of multiple non-Hispanic races, 65.6% were non-Hispanic White, and 5.5% were of unknown race or ethnicity. Overall, 1 423 084 enrollees (39.8%) received a booster. Veterans aged 18 to 34 years had the lowest booster rates vs veterans 85 years or older (Table 1 and Table 2). Veterans less likely to receive boosters included those not assigned vs assigned a primary care team, those with rural vs urban residence, and those reporting housing and/or food insecurity vs not. Veterans from the East South Central region had the lowest rates compared with those from New England (reference group). Black or African American veterans had the highest booster rates (44.3%); American Indian or Alaska Native veterans had the lowest (35.4%) (Table 1 and Table 2).

**Table 1.** COVID-19 Vaccine Booster Rates by Demographic, Clinical, and Social Determinants for Veterans Completing Primary Vaccination Series^a^

Characteristic	Veterans eligible to receive booster, No. (N = 3 578 627)	Veterans who received booster, No. (%) (n = 1 423 084 [39.8%])
Age, y		
18-34	145 522	21 533 (14.8)
35-49	410 445	92 747 (22.6)
50-64	826 576	317 605 (38.4)
65-74	1 116 654	524 877 (47.0)
75-84	777 762	354 316 (45.5)
≥85	301 668	112 006 (37.1)
Sex		
Men	3 260 197	1 310 836 (40.2)
Women	318 430	112 248 (35.3)
Urban or rural residence		
Urban or suburban	2 443 579	1 011 773 (41.4)
Rural or highly rural	1 135 048	411 311 (36.2)
Geographic regions by US Census Division^b^		
East North Central	419 724	194 974 (46.5)
East South Central	250 799	87 612 (34.9)
Middle Atlantic	312 052	140 148 (44.9)
Mountain	309 277	119 482 (38.6)
New England	149 339	68 256 (45.7)
Pacific	456 472	169 849 (37.2)
South Atlantic	898 526	340 251 (37.9)
West North Central	324 638	142 607 (43.9)
West South Central	457 800	159 905 (34.9)
Race and ethnicity		
American Indian or Alaska Native	21 462	7587 (35.3)
Asian	49 308	18 352 (37.2)
Hispanic	254 271	99 984 (39.3)
Native Hawaiian or other Pacific Islander	26 613	10 307 (38.7)
Non-Hispanic Black or African American	653 852	289 803 (44.3)
Non-Hispanic multiple race	28 267	10 362 (36.7)
Non-Hispanic White	2 346 630	918 268 (39.1)
Declined, unknown by patient, or missing	198 224	68 421 (34.5)
Disability		
Not miliary service connected	1 278 642	511 252 (40.0)
Miliary service connected	2 299 985	911 832 (39.6)
Food and/or housing insecurity^c^		
None (negative screen)	2 927 206	1 208 448 (41.3)
Present (positive screen)	182 076	71 244 (39.1)
Unknown	469 345	143 392 (30.5)
Primary care team assignment		
None	189 955	29 515 (15.5)
Assignment	3 388 672	1 393 569 (41.1)
No. of primary care visits in prior year		
0	233 336	54 898 (23.5)
1-2	1 078 948	302 174 (28.0)
3-5	1 276 382	526 362 (41.2)
≥6	989 961	539 650 (54.5)
No. of mental health visits in prior year		
0	2 534 745	993 696 (39.2)
1-2	307 830	120 856 (39.3)
3-5	302 318	125 036 (41.4)
≥6	433 734	183 496 (42.3)
Hospitalization or death probability in next year (CAN index), %^d^		
0-9	1 571 966	490 495 (31.2)
10-19	917 137	381 387 (41.6)
20-39	631 284	309 234 (49.0)
40-99	458 240	241 968 (52.8)
No. of mental health diagnoses		
0	2 325 364	914 032 (39.3)
≥1	1 253 263	509 052 (40.6)
Prior SARS-CoV-2		
Negative results before vaccination	1 033 697	506 927 (49.0)
≥1 Positive result before vaccination	122 487	51 606 (42.1)
No test results available	2 422 443	864 551 (35.7)

Abbreviation: CAN, Care Assessment Needs risk index.

^a^ Data are from date of Pfizer Emergency Use Authorization (EUA) approval (December 11, 2020) to February 8, 2022; some veterans may have participated in clinical trials before the EUA date but have completed 2 doses and are eligible for booster. Percentages are rounded and therefore may not total 100.

^b^ East North Central includes Illinois, Indiana, Michigan, Ohio, and Wisconsin; East South Central, Alabama, Kentucky, Mississippi, and Tennessee; Middle Atlantic, New Jersey, New York, and Pennsylvania; Mountain, Arizona, Colorado, Idaho, Montana, New Mexico, Nevada, Utah, and Wyoming; New England, Connecticut, Massachusetts, Maine, New Hampshire, Rhode Island, and Vermont; Pacific, Alaska, California, Hawaii, Oregon, and Washington; South Atlantic, Delaware, Florida, Georgia, Maryland, North Carolina, South Carolina, Virginia, Washington, DC, and West Virginia; West North Central, Iowa, Kansas, Minnesota, Missouri, North Dakota, Nebraska, and South Dakota; and West South Central, Arkansas, Louisiana, Oklahoma, and Texas.

^c^ Derived from a Veterans Health Administration patient screening tool administered before most but not all clinic visits.

^d^ The range is 0 to 99; higher scores indicate greater risk of hospitalization or death.

**Table 2.** Adjusted COVID-19 Vaccine Booster Rates and Rate Differences by Demographic, Clinical, and Social Characteristics for 3 578 627 Veterans Completing Primary Vaccination Series^a^

Characteristic	Adjusted rate, % (95% CI)^b^	ARD, % (95% CI)^c^
Age, y		
18-34	15.1 (14.9-15.3)	−22.0 (−22.2 to −21.7)
35-49	22.6 (22.4-22.7)	−14.5 (−14.8 to −14.3)
50-64	35.0 (34.9-35.1)	−2.1 (−2.3 to −1.8)
65-74	42.8 (42.7-42.9)	5.7 (5.5 to 5.9)
75-84	43.2 (43.1-43.3)	6.1 (5.9 to 6.3)
≥85	37.1 (36.9-37.3)	[Reference]
Sex		
Men	36.0 (36.0-36.1)	0.1 (−0.1 to 0.3)
Women	35.9 (35.8-36.1)	[Reference]
Urban or rural residence		
Urban or suburban	38.0 (37.9-38.1)	[Reference]
Rural or highly rural	32.1 (32.0-32.2)	−5.9 (−6.0 to −5.8)
Geographic regions by US Census Division		
East North Central	42.0 (41.9-42.2)	−0.8 (−1.1 to −0.5)
East South Central	31.0 (30.9-31.2)	−11.8 (−12.1 to −11.5)
Middle Atlantic	40.8 (40.6-40.9)	−2.1 (−2.3 to −1.8)
Mountain	35.5 (35.4-35.7)	−7.3 (−7.6 to −7.1)
New England	42.8 (42.6-43.1)	[Reference]
Pacific	35.0 (34.9-35.2)	−7.8 (−8.1 to −7.5)
South Atlantic	33.3 (33.2-33.4)	−9.6 (−9.8 to −9.3)
West North Central	41.4 (41.3-41.6)	−1.4 (−1.7 to −1.1)
West South Central	32.4 (32.3-32.5)	−10.5 (−10.7 to −10.2)
Race and ethnicity		
American Indian or Alaska Native	34.0 (33.4-34.6)	−0.6 (−1.2 to −0.0)
Asian	41.8 (41.3-42.2)	7.2 (6.7 to 7.6)
Hispanic (any race)	38.7 (38.5-38.9)	4.1 (3.9 to 4.3)
Native Hawaiian or other Pacific Islander	36.8 (36.2-37.3)	2.1 (1.6 to 2.7)
Non-Hispanic Black or African American	40.8 (40.7-40.9)	6.2 (6.0 to 6.3)
Non-Hispanic White	34.6 (34.6-34.7)	[Reference]
Multipe non-Hispanic races	36.7 (36.1-37.2)	2.0 (1.5 to 2.6)
Declined, unknown by patient, or missing	33.8 (33.6-34.0)	−0.8 (−1.1 to −0.6)
Disability		
Not miliary service connected	35.1 (35.0-35.1)	−1.5 (−1.6 to −1.4)
Military service connected	36.6 (36.5-36.6)	[Reference]
Food and/or housing insecurity		
None (negative screen)	36.9 (36.8-37.0)	[Reference]
Present (positive screen)	31.5 (31.3-31.6)	−5.4 (−5.6 to −5.3)
Unknown	32.7 (32.6-32.9)	−4.2 (−4.3 to −4.0)
Primary care team assignment		
None	16.3 (16.1-16.5)	−21.3 (−21.5 to −21.2)
Assignment	37.7 (37.6-37.7)	[Reference]
No. of primary care visits in prior year		
0	29.4 (29.1-29.6)	[Reference]
1-2	28.3 (28.2-28.4)	−1.1 (−1.3 to −0.9)
3-5	38.2 (38.1-38.3)	8.8 (8.6 to 9.1)
≥6	45.7 (45.6-45.8)	16.3 (16.1 to 16.6)
No. of mental health visits in prior year		
0	35.9 (35.8-35.9)	[Reference]
1-2	35.0 (34.8-35.1)	−0.9 (−1.1 to −0.7)
3-5	36.3 (36.2-36.5)	0.5 (0.3 to 0.6)
≥6	37.5 (37.4-37.7)	1.6 (1.5 to 1.8)
Hospitalization or death probability in next year (CAN index), %		
0-9	33.5 (33.4-33.6)	[Reference]
10-19	36.9 (36.8-37.0)	3.4 (3.2 to 3.5)
20-39	38.9 (38.8-39.1)	5.4 (5.3 to 5.6)
40-99	39.5 (39.4-39.7)	6.0 (5.8 to 6.2)
No. of mental health diagnoses		
0	36.3 (36.2-36.4)	[Reference]
≥1	35.5 (35.4-35.6)	−0.8 (−0.9 to −0.7)
Prior SARS-CoV-2		
Negative results before vaccination	39.4 (39.3-39.5)	[Reference]
≥1 Positive result before vaccination	34.8 (34.6-35.0)	−4.6 (−4.8 to −4.4)
No test results available	34.7 (34.7-34.8)	−4.7 (−4.8 to −4.6)

Abbreviation: ARD, adjusted rate difference.

^a^ Data are from date of Pfizer Emergency Use Authorization (EUA) approval (December 11, 2020) to February 8, 2022; some Veterans may have participated in clinical trials before the EUA date but have completed 2 doses and are eligible for booster.

^b^ Rates and rate differences are adjusted for all covariates in the Table.

^c^ Indicates rate difference compared with the reference group. Negative values indicate lower rates than the reference group and positive values indicate higher rates than the reference group. *P* < .001 for all comparisons except sex (men vs women, *P* = .29).

## Discussion

 This cohort study found that less than half of eligible US veterans have received a COVID-19 vaccination booster. Low booster rates in a population with primary vaccination is concerning; combined with those never vaccinated, millions are susceptible to COVID-19–related illness, hospitalization, and mortality. At greatest risk are veterans who are younger, are from American Indian or Alaska Native populations, reside in the South or in rural areas, are not assigned a primary care team, and report housing and/or food insecurity. Whereas Black and African American individuals in the general population are less likely to be vaccinated,^3^ the opposite occurred in the VHA because the VHA system has fewer barriers to access.^4,5^ Therefore, these results may not generalize to nonveteran populations, and the VHA EHR may not capture all community-administered boosters. Nevertheless, the VHA serves more than 6 million US residents each year. Outreach to younger, rural, American Indian or Alaska Native, and homeless populations and encouragement of primary care clinicians to engage unvaccinated and unboosted patients in conversations about COVID-19 vaccination^6^ may mitigate residual disparities.

## Notes

Supplement. **eMethods.** Statistical Analysis **eFigure.** Study Population Derivation
