# Supplementary material for: Association of Demographic, Clinical, and Social Determinants of Health With COVID-19 Vaccination Booster Dose Completion Among US Veterans
Source: JAMA Netw Open. 2022 Jul 19;5(7):e2222635. doi: 10.1001/jamanetworkopen.2022.22635 (PMC9297115; doi:10.1001/jamanetworkopen.2022.22635)
Supplement: Supplement. — eMethods. Statistical Analysis eFigure. Study Population Derivation [file jamanetwopen-e2222635-s001.pdf]

Supplementary Online Content

Seal KH, Bertenthal D, Manuel JK, Pyne JM. Association of demographic, clinical, and social determinants of health with COVID-19 vaccination booster dose completion among US veterans. *JAMA Netw Open*. 2022;5(7):e2222635. doi:10.1001/jamanetworkopen.2022.22635

eMethods. Statistical Analysis
eFigure. Study Population Derivation

This supplementary material has been provided by the authors to give readers additional information about their work.

© 2022 Seal KH et al. *JAMA Network Open*.

eMethods. Statistical Analysis

All covariates (subgroups) are listed in Tables 1 and 2. Data were prepared using SAS 9.4 and analyzed using Stata 17.0.

With the descriptive focus of this analysis, we reported generalized linear models as adjusted rates and adjusted rate differences. The objective of modeling was to deconfound rate differences between subgroups of interest. What is important are the magnitudes of the differences, which need to be interpreted in clinical and policy context, compared with hypothesis testing in which the focus would be whether group differences excluded the null, and issues surrounding Type I and Type II error would be important. As such, the confidence intervals provide an indication of model precision and for that purpose we opted for a traditional alpha of 0.05 to construct two-sided confidence intervals.

© 2022 Seal KH et al. *JAMA Network Open*.

eFigure. Study Population Derivation

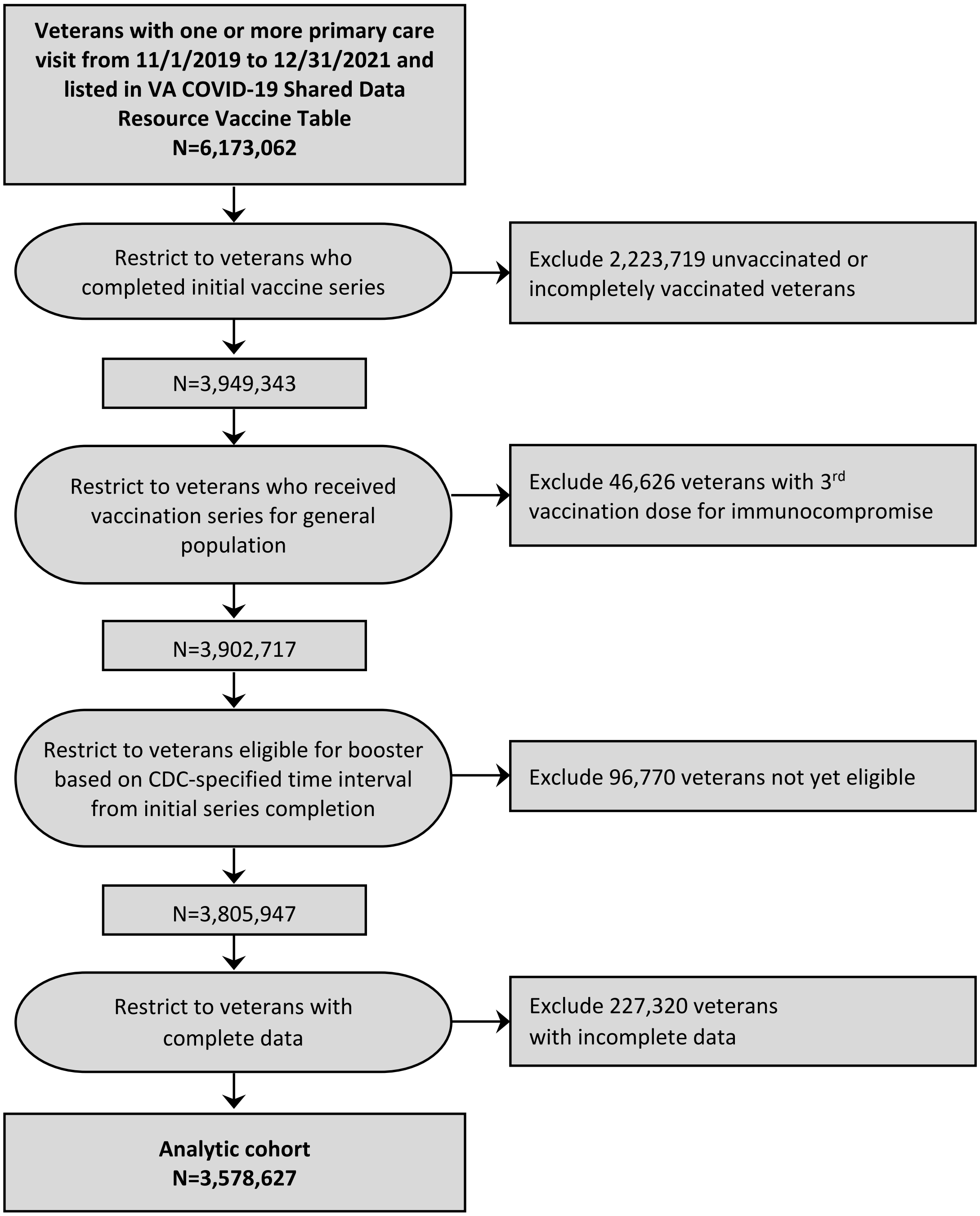

© 2022 Seal KH et al. *JAMA Network Open*.
